# DAT genotype modulates striatal processing and long-term memory for items associated with reward and punishment^☆^

**DOI:** 10.1016/j.neuropsychologia.2013.07.018

**Published:** 2013-09

**Authors:** Bianca C. Wittmann, Geoffrey C. Tan, John E. Lisman, Raymond J. Dolan, Emrah Düzel

**Affiliations:** a Wellcome Trust Centre for Neuroimaging, University College London, London, WC1N 3BG, UK; b Helen Wills Neuroscience Institute, University of California, Berkeley, CA 94720, USA; c Department of Psychology, University of Giessen, 35394 Giessen, Germany; d Psychiatry Residency Programme, National Healthcare Group/Ministry of Health, Singapore, Singapore; e Agency for Science, Technology and Research, Singapore, Singapore; f Department of Biology and Volen Center for Complex Systems, Brandeis University, Waltham, MA 02454, USA; g Institute of Cognitive Neuroscience, University College London, London, W1CN 4AR, UK; h Institute of Cognitive Neurology and Dementia Research, Otto-von-Guericke-University, 39120 Magdeburg, Germany; i German Centre for Neurodegenerative Diseases (DZNE), Standort, Magdeburg, Germany

**Keywords:** Episodic memory, Reward, Dopamine, Hippocampus, Striatum

## Abstract

Previous studies have shown that appetitive motivation enhances episodic memory formation via a network including the substantia nigra/ventral tegmental area (SN/VTA), striatum and hippocampus. This functional magnetic resonance imaging (fMRI) study now contrasted the impact of aversive and appetitive motivation on episodic long-term memory. Cue pictures predicted monetary reward or punishment in alternating experimental blocks. One day later, episodic memory for the cue pictures was tested. We also investigated how the neural processing of appetitive and aversive motivation and episodic memory were modulated by dopaminergic mechanisms. To that end, participants were selected on the basis of their genotype for a variable number of tandem repeat polymorphism of the dopamine transporter (DAT) gene. The resulting groups were carefully matched for the 5-HTTLPR polymorphism of the serotonin transporter gene. Recognition memory for cues from both motivational categories was enhanced in participants homozygous for the 10-repeat allele of the DAT, the functional effects of which are not known yet, but not in heterozygous subjects. In comparison with heterozygous participants, 10-repeat homozygous participants also showed increased striatal activity for anticipation of motivational outcomes compared to neutral outcomes. In a subsequent memory analysis, encoding activity in striatum and hippocampus was found to be higher for later recognized items in 10-repeat homozygotes compared to 9/10-repeat heterozygotes. These findings suggest that processing of appetitive and aversive motivation in the human striatum involve the dopaminergic system and that dopamine plays a role in memory for both types of motivational information. In accordance with animal studies, these data support the idea that encoding of motivational events depends on dopaminergic processes in the hippocampus.

## Highlights

• Effect of reward and punishment on episodic memory is modulated by DAT genotype.
• Enhanced recognition memory for motivational cues in DAT 10-repeat homozygotes.
• Higher activation at encoding in striatum and hippocampus in DAT 10-repeat homozygotes.

## 1 Introduction

Reward improves episodic memory formation in humans (Shohamy & Adcock, 2010). Functional imaging studies have shown that memory encoding of reward-associated stimuli involves a network of dopaminergic midbrain areas, ventral striatum and hippocampus (Adcock, Thangavel, Whitfield-Gabrieli, Knutson, & Gabrieli, 2006; Callan & Schweighofer, 2008; Krebs, Schott, Schutze, & Duzel, 2009; Wittmann et al., 2005). Evidence from animal studies suggests that this reward-related modulation of long-term memory could be mediated by dopamine release in the hippocampus (Bethus, Tse, & Morris, 2010; for a review of dopamine effects on hippocampal long-term potentiation, see Lisman, Grace, & Duzel, 2011; Otmakhova, Duzel, Deutch, & Lisman, 2013; Rossato, Bevilaqua, Izquierdo, Medina, & Cammarota, 2009). This is supported by studies in humans indicating that dopamine binding potential in the hippocampus is correlated with memory performance (Backman et al., 2000; Cervenka, Backman, Cselenyi, Halldin, & Farde, 2008; Takahashi et al., 2007, 2008).

In contrast to the memory effects of monetary reward, little is known about the effects of monetary punishments on episodic memory formation. For emotional stimuli, it has been shown that negative emotional events are remembered better than emotionally neutral events, and that this effect involves the amygdala (for a review see Murty, Ritchey, Adcock, & LaBar, 2010). For aversive motivation, there have been inconsistent reports across a range of human memory tasks. Whereas aversive electrical stimulation impaired memory in a human version of the Morris water maze (Murty, LaBar, Hamilton, & Adcock, 2011), threat of shocks enhanced memory for scene images when participants were tested 24 h later, an effect that was based on amygdala-hippocampal interaction at encoding (Murty, LaBar, & Adcock, 2012). When monetary rewards and punishments were dependent on memory performance, threat of monetary loss enhanced source memory retrieval in a similar manner to reward when tested immediately after learning (Shigemune, Tsukiura, Kambara, & Kawashima, 2013). This was associated with a correlation of activity in striatum and hippocampus during successful source retrieval. In contrast, punishment cues during an incidental memory task had no effect on item recollection or recognition when tested immediately after learning (Mather & Schoeke, 2011). These contrasting results suggest that the effect of punishment on memory may be dependent on contextual influences. The current study investigated whether monetary punishment affects memory consolidation through a dopaminergic network.

Appetitive and aversive motivation have been suggested to be processed in opponent brain systems, with rewards eliciting dopaminergic activity and punishments eliciting serotonergic activity (Daw, Kakade, & Dayan, 2002). More recent data indicate that punishments can also induce firing of dopaminergic neurons in rats (Brischoux, Chakraborty, Brierley, & Ungless, 2009) and monkeys (Bromberg-Martin, Matsumoto, & Hikosaka, 2010; Matsumoto & Hikosaka, 2009), although other data suggest that punishment-responsive SN/VTA neurons are GABAergic (Cohen, Haesler, Vong, Lowell, & Uchida, 2012). By combining fMRI with genetics, the current study investigated transmitter specificity of midbrain signals in humans. In humans, striatal activity has been shown to correlate with aversive predictions (Carter, Macinnes, Huettel, & Adcock, 2009; Delgado, Li, Schiller, & Phelps, 2008; Seymour et al., 2004; Seymour, Daw, Dayan, Singer, & Dolan, 2007). Current models propose that the interaction of appetitive and aversive motivation in the dopamine and serotonin systems could depend on the overall motivational value of the context and on action requirements (Boureau & Dayan, 2011; Cools, Nakamura, & Daw, 2011). A recent study supports these models by demonstrating the relevance of action requirements for activation of SN/VTA and the striatum in humans (Guitart-Masip et al., 2011). Thus, when investigating the effects of dopamine-related polymorphisms on episodic memory for appetitive and aversive events, it is important to stratify and match populations for polymorphisms that influence serotonergic neurotransmission. After non-synaptic sources, transporter concentration is the most important factor in neurotransmitter homeostasis (Pendyam, Mohan, Kalivas, & Nair, 2012). The genes for the serotonin transporter, SLC6A4/SERT, and the dopamine transporter, SLC6A3/DAT1, both contain length variations in their promoter regions that regulate expression of their respective transporters. As transporters both influence speed of reuptake from the synapse and increase presynaptic neurotransmitter availability, they may be expected to shape phasic neuromodulation seen in reward and punishment.

The current study investigated (i) whether anticipation of monetary punishments modulates episodic memory, (ii) whether reward and punishment related anticipation and memory are modulated by dopamine transporter genotype under conditions when groups are matched for serotonin transporter genotype, and (iii) the common and dissociable fMRI correlates of these processes. Subjects were genotyped for common polymorphisms in the dopamine transporter (DAT1 VNTR) and serotonin transporter (5-HTTLPR) and scanned during a motivational anticipation task, followed one day later by a memory test outside the scanner. In line with previous studies (Adcock et al., 2006; Wittmann et al., 2005; Wittmann, Schiltz, Boehler, & Duzel, 2008), we expected reward-predicting stimuli to activate the SN/VTA system and enhance episodic memory. Based on reports of activations in the dopaminergic system for aversive stimuli, we hypothesized that punishment prediction would also activate the mesolimbic system. Increased dopaminergic transmission was expected to lead to improved episodic memory performance.

## 2 Experimental procedures

### 2.1 Participants

A total of 24 healthy adults (all right-handed, mean age [±SD] 25.3±3.9 years; 8 men) participated in the study. They were screened for neurological conditions and past psychiatric disorders using the Mini International Neuropsychiatric Inventory (Sheehan et al., 1998) and provided blood samples for genotyping. The study was designed to compare DAT 9-repeat carriers and 10-repeat homozygotes based on previous reports of a role of the DAT VNTR in dopaminergic modulation of memory (Bertolino et al., 2008; Schott et al., 2006). We here report comparisons of the DAT 10-repeat homozygotes with DAT 9/10-repeat heterozygotes. There were no DAT 9-repeat homozygotes in the participant sample. The two DAT groups were matched for age, gender and 5-HTTLPR genotype. In relation to 5-HTTLPR, only short allele homozygotes (SS) and long allele homozygotes (LL) were included in this study. The final sample included 12 participants from each DAT group. Half of the participants in each group were SS homozygotes and half were LL homozygotes. The majority of participants were invited based on their genotype. Additionally, some participants were genotyped after scanning and excluded if they were heterozygous for 5-HTTLPR (seven participants). Because the overall sample was non-random, we did not calculate Hardy–Weinberg equilibrium statistics. Twenty-one participants were Caucasian, three participants were Asian (two 10-repeat homozygotes, one 9/10-repeat heterozygous). To address possible effects of ethnicity, second-level analyses were performed excluding the three Asian participants. Since there was no significant change in the result, we report analyses of the combined group. All participants gave written informed consent, and the study was approved by the local ethics committee.

### 2.2 Behavioural task

We obtained fMRI data while participants were completing alternating blocks of a reward and punishment task (modified from Wittmann et al., 2005). Before entering the scanner, participants received written instructions and completed a practice version of each block type. The anticipation task was presented in alternating blocks of reward and punishment. In each block, motivational stimuli were randomly mixed with neutral stimuli in an event-related design. This design allowed a contrast of each motivational category with corresponding neutral items from the same block as well as a direct contrast of appetitive and aversive motivational processes. At the beginning of each block, participants were informed of the motivational block type (reward/punishment). At the beginning of each trial, the motivational status of the trial (motivational/neutral) was indicated by the category of a picture on the screen (indoor/outdoor scene). In motivational trials, participants were rewarded or punished for their performance on a rapid number detection task. One day after scanning, participants performed a recognition memory task on the cue pictures.

**Fig. 1:**
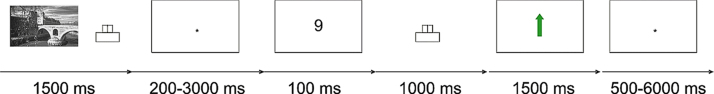
Experimental design. Trial sequence for the study phase, exemplified for a rewarded trial from a reward block. A cue picture was presented indicating whether participants could win money on that trial. Participants made a category decision on the picture, waited for the following number task, and then indicated quickly whether the number was higher or lower than five. In rewarded trials, they received “win” feedback after correct decisions made within a time limit. In neutral trials, they did not receive meaningful feedback. In punishment blocks, the cue category predicted punishment or neutral outcomes.

On day 1, participants engaged in three fMRI sessions of 8–9 min length, each consisting of one reward block followed by one punishment block (Fig. 1). Each block contained 38 (reward blocks) or 32 (punishment blocks) trials of 4.3–11.1 s duration, half of which were potentially rewarded/punished. Picture category (indoor or outdoor) indicated the motivational status of each trial. One category predicted neutral trials (neither reward nor punishment). The other category predicted reward in the reward blocks and punishment in the punishment blocks. Data from the first three motivational and first three neutral trials of each block were discarded to allow for switching effects. Additionally, the last six trials in reward blocks were discarded to eliminate a potential confound of different block lengths, which were necessary to ensure overall monetary gain for participants. During each trial, participants saw a greyscale landscape photograph for 1500 ms, responded to it with a button press (right index or middle finger) indicating the motivational status of the trial (reward/neutral in reward blocks, punishment/neutral in punishment blocks), waited a variable interval (delay, 200–3000 ms duration), and then responded to a number (target, 100 ms) by button press. Visual feedback (1000 ms duration) was given 1000 ms after presentation of the target. A variable fixation phase (500–4500 ms) followed. The speeded number comparison task (Wittmann et al., 2005) required participants to decide whether the target number (1, 4, 6 or 9) was lower or higher than 5. They responded as quickly as possible by button press with their right index or middle finger. A response time limit was used to determine trial outcome.

In reward trials, participants received no-win feedback (£0, yellow downward arrow) if their response to the target number was incorrect or exceeded the response time limit. After correct decisions within the time limit, they received win feedback (£1, green upward arrow). In punishment trials, participants received loss feedback (−£1, red downward arrow) if their response to the target number was incorrect or exceeded the response time limit. After correct decisions within the time limit, they received no-loss feedback (£0, yellow upward arrow). The time limit was adjusted individually in a staircase procedure to ensure reward and punishment rates of ~66%. In neutral trials, uninformative feedback was given. Participants were informed of the speed-accuracy requirements and cue categories. Frequency of target buttons and numbers was counterbalanced for each session. Participants were asked to pay attention to the cues to ensure awareness of the reward/punishment status of each trial, but not told that a memory test would follow.

In the memory test given one day after the study session, participants were shown all images from the study phase randomly mixed with newly presented distractor images. Participants received written instructions and additional examples detailing the difference between ‘remember’ and ‘know’ responses. First, participants indicated whether they recognized the image (‘Old/New’). For images classified as old, they then distinguished between recollection and familiarity according to the remember/know procedure (‘Remember/Know/Guess’) (Düzel, Yonelinas, Mangun, Heinze, & Tulving, 1997; Tulving, 1985). For images classified as new, participants indicated whether their decision was confident (‘Sure/Guess’). Response time limits were set at 3 s for each decision. A fixation phase of 1.5 s followed. Every 96 trials, the task was paused until participants were ready to continue.

### 2.3 Behavioural analysis

Participants’ reaction times and hit rates during the study task were analysed in repeated-measures ANOVAs. Adding the remember and know rates obtained in the memory test for old stimuli (percentage of studied items classified as remembered or known) and subtracting the corresponding false alarm rate for distractors (percentage of unstudied items classified as remembered or known) yielded corrected hit rates. We also calculated a corrected remember rate and a corrected know rate separately by subtracting the corresponding false alarm rates. Note that these response rates excluded trials in which participants guessed.

### 2.4 fMRI acquisition

Magnetic resonance images were acquired on a 3 T Allegra head scanner (Siemens Medical Systems, Erlangen, Germany) with a head coil for RF transmission and signal reception. A field map was acquired with a double echo gradient echo field map sequence (TE, 10.0 and 12.46 ms; TR, 1020 ms; matrix size, 64×64), using 64 slices covering the whole head (voxel size, 3×3×3 mm), to improve distortion correction of the functional images. For functional images, we used BOLD signal sensitive T2⁎-weighted transverse single-shot gradient-echo echo-planar imaging (EPI). Each volume contained 35 slices of 1.5 mm thickness and 1.5 mm in-plane resolution (TR 3.5 s, TE 30 ms, matrix size, 128×128). Coverage was obtained from the base of the orbitofrontal cortex and the medial temporal lobe (MTL) to the superior border of the anterior cingulate cortex. Possible BOLD sensitivity losses in the hippocampus due to susceptibility artifacts were minimized by applying a *z*-shim gradient moment of 0.6 mT m^−1^ ms^−1^ and a slice orientation of −30° to the AC-PC line (Weiskopf, Hutton, Josephs, & Deichmann, 2006). For normalization, a whole-brain image (100 slices) with the same EPI parameters was used. In each scanning session, ~150 functional whole brain volumes were acquired. Scanner noise was reduced with ear plugs, and participants’ head movements were minimized with foam pads. Additionally, anatomical scans were collected using multi-echo 3D FLASH for mapping proton density, T1 and magnetization transfer (MT) at 1 mm resolution (Helms, Draganski, Frackowiak, Ashburner, & Weiskopf, 2009; Weiskopf & Helms, 2008).

### 2.5 fMRI analysis

Preprocessing and data analysis were performed using Statistical Parametric Mapping software implemented in Matlab (SPM5; Wellcome Trust Center for Neuroimaging, London, UK). Using the FieldMap toolbox (Hutton et al., 2002; Hutton, Deichmann, Turner, & Andersson, 2004), field maps were estimated from the phase difference between the images acquired at the short and long TE. The EPI images were corrected for distortions based on the field map (Hutton et al., 2002) and the interaction of motion and distortion using the Unwarp toolbox (Andersson, Hutton, Ashburner, Turner, & Friston, 2001; Hutton et al., 2004). EPI images were then spatially normalized to the Montreal Neurological Institute (MNI) template by warping the acquired whole-head EPI to the SPM template and applying these parameters to the functional images (voxel size 1×1×1 mm), and smoothed using a 4 mm Gaussian kernel. A high-pass filter with a cutoff of 128 s was applied to the data.

For statistical analysis, trial-related activity for each participant was assessed by convolving a vector of trial onsets with a canonical hemodynamic response function (Friston et al., 1998). A general linear model (GLM) was specified for each participant to model the effects of interest and six covariates capturing residual motion-related artefacts. After creating statistical parametric maps for each participant by applying linear contrasts to the parameter estimates, a random effects analysis was performed to assess group effects. The relevant contrasts were: Reward-predicting vs. neutral cue, punishment-predicting vs. neutral cue, reward-predicting vs. punishment-predicting cues and the reverse contrast. We also analyzed activity in the encoding phase with respect to subsequent memory performance on a trial-by-trial basis (difference due to memory, dm) for recognised vs. forgotten rewarded, punished and neutral items.

The statistical threshold for the imaging results was set to *p*<0.05, family-wise error (FWE) rate corrected for spherical search volumes in pre-defined areas. The areas of interest were chosen based on experimental results from the reward-based memory paradigm: The putamen and substantia nigra were chosen based on Wittmann et al. (2005, 2008), the anterior hippocampus was chosen based on Wittmann et al. (2005) and the amygdala was chosen based on Wittmann et al. (2008) and because of its relevance for aversive memory formation (Murty et al., 2010). Spherical SVC was centered on peak voxels identified in these regions. The radius of each SVC corresponded to the anatomical volumes of the a priori regions as reported in anatomical studies. These were: 9 mm for activations in the ventral striatum (see Anastasi et al., 2006), 6 mm for activations in the anterior hippocampus (see Lupien et al., 2007), 7.5 mm for activations in the amygdala (see Brierley, Shaw, & David, 2002) and 4.5 mm for activations in the substantia nigra (see Geng, Li, & Zee, 2006). Activations are displayed at a threshold of *p*<0.005 (uncorrected) with 15 contiguous voxels unless stated otherwise. All stereotaxic coordinates are given in MNI space. All brain images are shown in neurological orientation. All behavioural averages are given as mean values±SE.

To better localize SN/VTA activity, relevant activation maps were superimposed on the mean image of the spatially normalized MT maps. MT imaging is based on the transfer of energy between protons in free water and highly bound protons within macromolecules (Wolff & Balaban, 1989). Thus MT saturation is thought to be a more direct measure to image myelin and improves contrast between SN and surrounding white matter tracts (Helms et al., 2009) without the geometric distortion present in iron-based imaging such as susceptibility and R2^⁎^ mapping. It has been shown to allow distinguishing the SN from surrounding structures as a bright area, which has been confirmed to be coextensive with the SN as delineated histologically by tyrosine hydroxylase immunohistochemistry (Bolding et al., 2013). It has also been shown to provide a measure of nigral degeneration in clinical populations such as Parkinson’s disease (Eckert et al., 2004; Tambasco et al., 2011). However, we will refer to BOLD activity from the entire SN/VTA complex throughout this paper because dopamine neurons are dispersed throughout the SN/VTA complex and form a functional continuum in primates (Düzel et al., 2009). This is underlined by recordings showing that dopamine neurons in the SN and VTA respond to reward (Ljungberg, Apicella, & Schultz, 1992; Tobler, Dickinson, & Schultz, 2003).

### 2.6 Genotyping

DNA was extracted from blood samples and genotyped by gene sizing. Primer sequences were chosen based on (Kang, Palmatier, & Kidd, 1999) and (Heils et al., 1996) and were checked on on Primer3 software (Rozen & Skaletsky, 2000) found and blasted by electronic polymerase chain reaction (PCR) on the UCSC genome browser NCBI build 36.1 (Karolchik et al., 2008).

**Table:** 

5-HTTLPR
Forward: 5′ HEX-GCGTTGCCGCTCTGAAT-3′
Backward: 5′-GGATGCGGGGGAATACTG-3′

DATVNTR (Cook et al., 1995; Kang et al., 1999)
Forward: 5′ HEX-TGTGGTGTAGGGAACGGCCTGAG-3′
Backward: 5′-CTTCCTGGAGGTCACGGCTCAAGG-3′

Genotyping was performed through PCR followed by restriction digest and subsequent capillary electrophoresis. PCR with Taq polymerase (Molzym) involved initial denaturation at 94 °C for 5 min, followed by 35 cycles of denaturation at 94 °C for 30 s, annealing at 61 °C for 30 s and elongation at 72 °C for 60 s, followed by 72 °C for 7 min. This was heat denatured to single-stranded fragments in formamide and run with a ROX500 ladder on a 3730xl DNA Analyser (Applied Biosystems). Individual genotypes were called according to peak size on GeneMapper software version 4.0.

## 3 Results

### 3.1 Behavioural effects

Participants successfully categorized the motivational and neutral cues (mean hit rate neutral: 96±1%, hit rate reward-predicting: 95±1%, hit rate punishment-predicting: 95±1%). As expected, a one-way repeated-measures ANOVA (three motivational levels and two between-subjects factors for 5-HTT and DAT genotype) on reaction times (RT) in the picture category task revealed a main effect of motivation (*F*_2,40_=5.9, *p*<0.01). Post-hoc one-tailed *t*-tests confirmed shorter RTs for reward-predicting (*t*_23_=2.1, *p*<0.05) and punishment-predicting (*t*_23_=4.1, *p*<0.001) compared to neutral cues (mean RT reward 673±38 ms, punishment 655±27 ms, neutral 722±26 ms).

There was a significant main effect of motivation on RTs to the number targets (*F*_2,40_=23.5, *p*<0.001) and no effect of or interaction with DAT genotype. Post-hoc one-tailed *t*-tests confirmed shorter RTs for reward (*t*_23_=2.1, *p*<0.05) and punishment trials (*t*_23_=4.0, *p*<0.001) compared to neutral trials (mean RT reward 431±18 ms, punishment 432±21 ms, neutral 506±21 ms). In the number task, rates of accuracy/reinforcement differed slightly from the targeted 0.67 rate because of participants’ effort in the two motivational categories (mean rate of win feedback in reward trials 0.7±0.05; neutral correct [in the absence of feedback for participants] 0.67±0.004; rate of loss feedback in punishment trials 0.65±0.003).

**Fig. 2:**
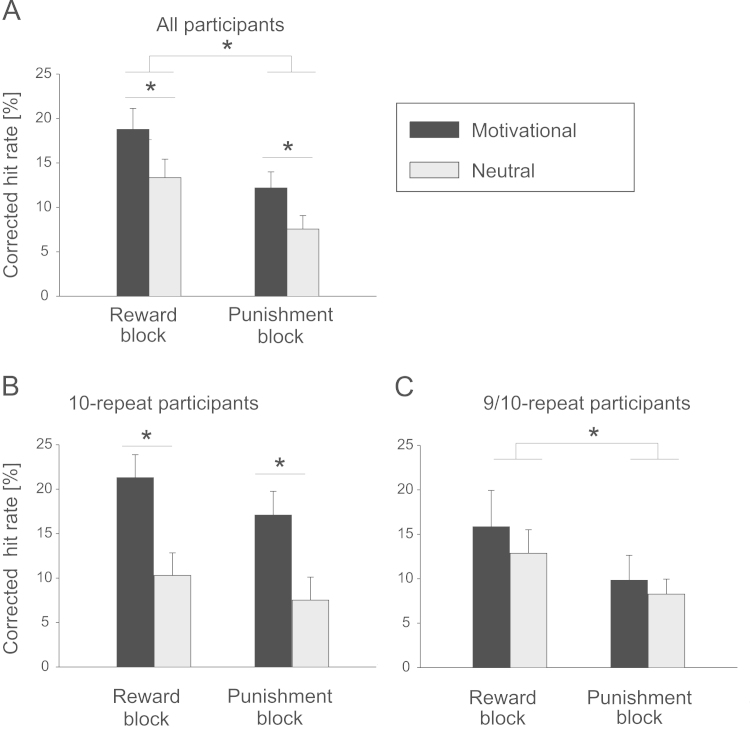
Memory performance on day 2 for cue pictures from the motivational task. (A) Mean recognition memory hit rates (±SE) across all participants, (B) mean hit rates (±SE) of DAT 10-repeat homozygous participants, (C) mean hit rates (±SE) of DAT heterozygous participants.

A previous study reported an effect of motivational outcome on memory for the cue (Mather & Schoeke, 2011). Since there was no memory difference based on motivational outcomes in the current study, trials were grouped for analysis based on cue type only. For the delayed memory test (Fig. 2A), a three-way ANOVA (reward/punishment block, motivational/neutral trials, remember/know responses) with two between-subjects factors (5-HTT and DAT genotype) revealed that memory performance was better for pictures from reward blocks than for pictures from punishment blocks (main effect of block; *F*_1,20_=14.5, *p*<0.01) and better for motivational (rewarded or punished) items than neutral items (main effect of motivation; *F*_1,20_=5.7, *p*<0.05). Based on our hypotheses, we performed post-hoc one-tailed paired t-tests on memory performance for rewarded and punished items compared to neutral items from the same block. Memory performance was significantly better for rewarded items compared to neutral items (*t*_23_=2.0, *p*<0.05) and for punished items compared to neutral items (*t*_23_=2.2, *p*<0.05). However, tested across all participants, punished items were not recognized better than neutral items from the reward block (*t*_23_=−0.4, *p*>0.5) and worse than rewarded items (*t*_23_=−2.2, *p*<0.05).

ANOVA results also showed interactions between memory performance and genotype. There was a significant interaction of DAT genotype with block, motivational status and remember/know judgment (*F*_1,20_=4.8, *p*<0.05). We then performed separate three-way ANOVAs on DAT 10-repeat homozygotes (Fig. 2B) and on 9/10-repeat heterozygotes (Fig. 2C) to explore this interaction effect. There was a main effect of motivation in 10-repeat homozygotes (*F*_1,11_=21.2, *p*<0.01) but not in 9/10-repeat heterozygotes (*F*_1,11_=0.1, *p*=0.76). Post-hoc paired *t*-tests on homozygous participants confirmed that memory for rewarded items was higher than for neutral items from reward blocks (*t*_11_=3.8, *p*<0.01) and memory for punished items was higher than for neutral items from punishment blocks (*t*_11_=3.0, *p*<0.05). In contrast to memory effects of the whole group, memory for punishment cues in 10-repeat homozygotes was also significantly better than memory for neutral items from the reward block (*t*_11_=2.4, *p*<0.05). In addition, the ANOVA showed a main effect of block in 9/10-repeat heterozygotes (*F*_1,11_=17.8, *p*<0.01) that only achieved trend-level significance in 10-repeat homozygotes (*F*_1,11_=3.7, *p*=0.08). Post-hoc paired t-tests revealed that items from the reward block were remembered better than items from the punishment block in 9/10-repeat heterozygotes (*t*_11_=3.7, *p*<0.01) but not in 10-repeat homozygotes (*t*_11_=1.9, *p*=0.09). There was no effect of remember/know judgment in either of the DAT groups. There was no effect of 5-HTTLPR on overall memory performance for punishment cues.

### 3.2 fMRI results

**Fig. 3:**
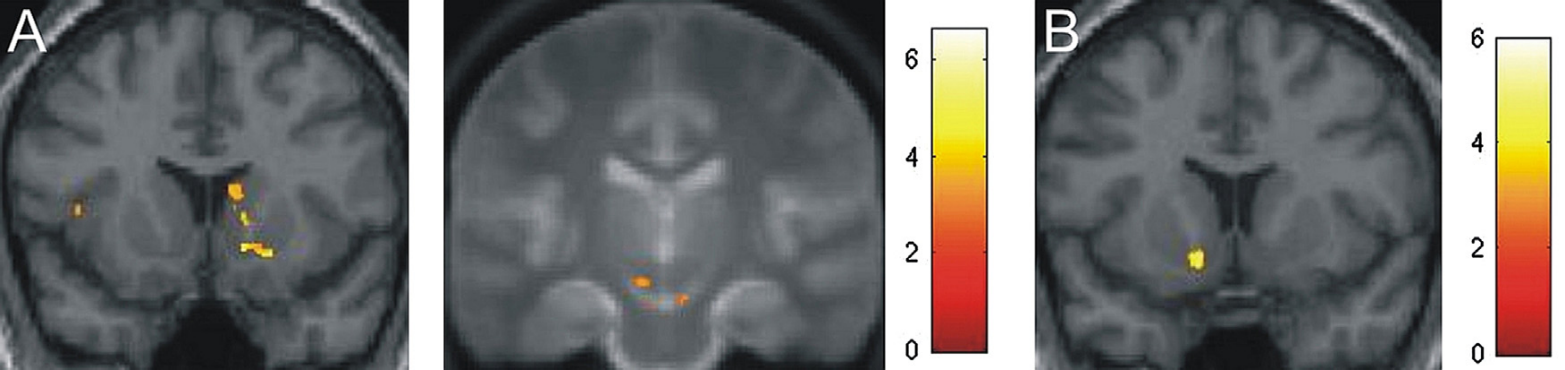
Neural response to anticipation of reward and punishment. (A) Overlapping activations in right ventral striatum and right SN/VTA to anticipation of rewards and punishments compared to neutral cues, displayed by inclusive masking of the reward anticipation contrast with the punishment anticipation contrast (SVC *p*<0.05). Peak voxels in striatum: 23, 14, −10 and 13, 14, −7; peak voxel in SN/VTA: 6, −21, −17. To better localize SN/VTA activations, the right panel displays an overlay onto an MT image (cf. methods section). (B) Stronger activation of the left ventral striatum for anticipation of rewards vs. anticipation of punishments (SVC *p*<0.05). Peak voxel: −11, 11, −10. Images are shown in neurological orientation at *p*<0.005, uncorrected, *k*>15 voxels, for visualization purposes. Peak coordinates are given in MNI space. Colour bars indicate *t* values.

Reward and punishment anticipation elicited overlapping activations in the SN/VTA-striatal system, as shown by inclusive masking of the reward anticipation contrast with the punishment anticipation contrast (Fig. 3A, Table 1). A direct contrast of reward-predicting vs. punishment-predicting pictures revealed a small cluster in the left ventral striatum (Fig. 3B), whereas the reverse contrast did not reveal any significant activations. At outcome time, there was no difference in activations in our a priori regions between reward and punishment.

**Fig. 4:**
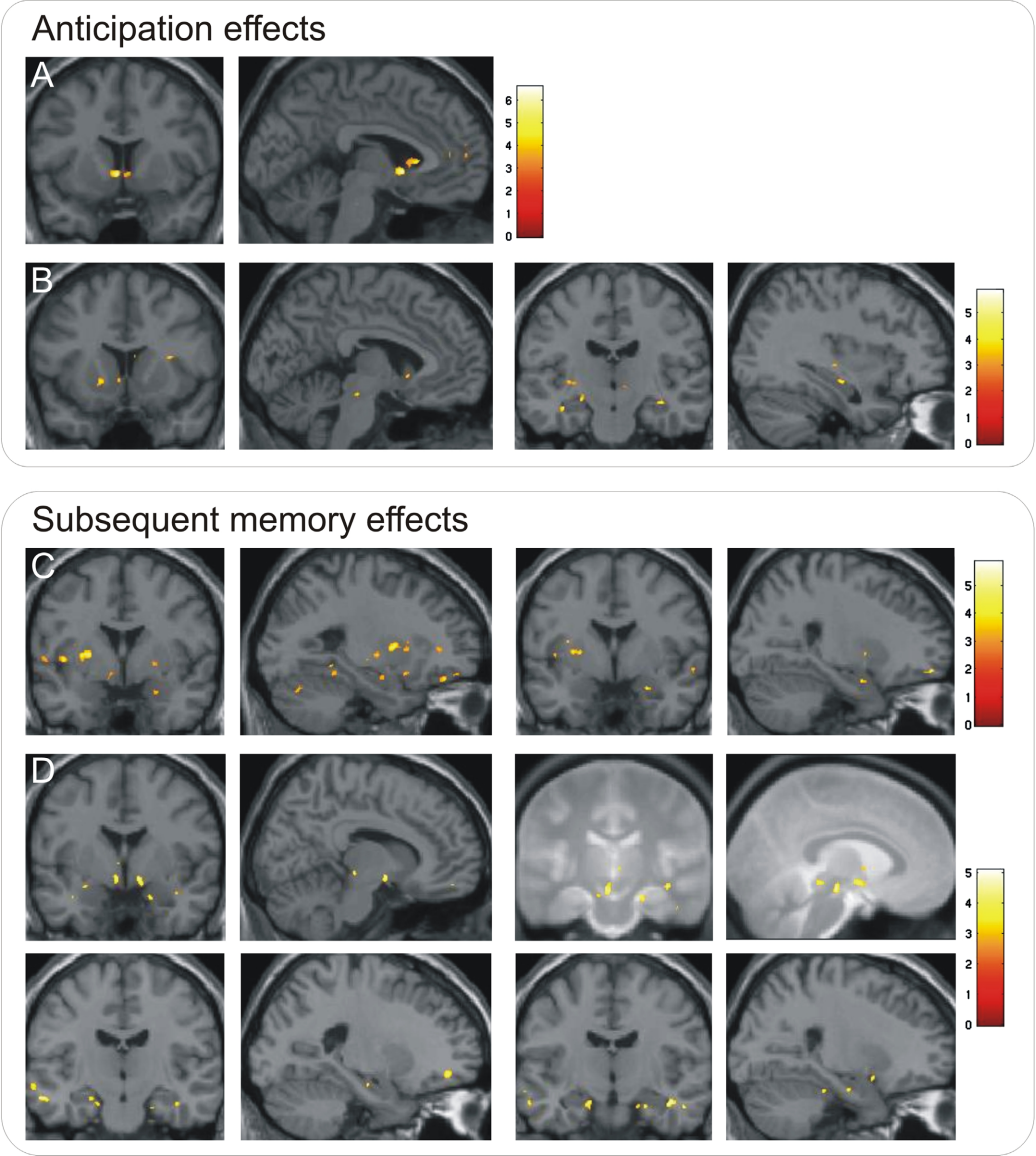
DAT genotype effects on neural responses. Stronger activations in DAT 10-repeat homozygotes compared to 9/10-repeat heterozygotes (all *p*<0.05 SVC). (A) Striatal activation to anticipation of rewards compared to neutral cues. Peak voxel: −3, 9, −2. (B) Striatal and hippocampal activations to anticipation of punishments compared to neutral cues. Peak voxel in striatum: 4, 13, −3; peak voxel in hippocampus: 36, −16, −13. (C) Striatal and hippocampal activations for subsequently recognized reward-predicting items. Peak voxel in striatum: −18, 16, 11 (no SVC correction); peak voxel in hippocampus: 25, −1, −21. (D) Striatal, midbrain and hippocampal activation for subsequently recognized punishment-predicting items. Peak voxel in striatum: −5, −2, −9 and 11, −1, −9 (top left panels); peak voxel in SN/VTA: −4, −20, −14 (top right panels); peak voxels in hippocampus (bottom panels): left: −15, −11, −20; right: 25, −11, −23. Images are displayed in neurological orientation at *p*<0.005, uncorrected, *k*>10 voxels, for visualization purposes. Peak coordinates are given in MNI space. Colour bars indicate t values. To better localize SN/VTA activations, the two corresponding panels display an overlay onto an MT image (cf. methods section).

Brain activity in response to both reward and punishment was influenced by genotype. DAT genotype affected responses to motivational items and motivational memory encoding. In comparison with 9/10-repeat heterozygotes, participants homozygous for the 10-repeat allele showed increased striatal activations for motivational compared to neutral cues (Fig. 4A and B). For punishment-predicting cues, activity was also higher in right hippocampus (Fig. 4B). Activity related to subsequent memory for reward-predicting items was higher in striatum and anterior hippocampus for homozygous participants compared to heterozygous participants (Fig. 4C). Later memory for punishment-predicting items was associated with activation of SN/VTA and bilateral hippocampus (Fig. 4D). The reverse contrasts revealed no significantly higher activations in heterozygotes compared homozygotes.

## 4 Discussion

These results suggest that the dopaminergic system is involved in the neural processing of appetitive and aversive motivation in humans and in memory formation for motivational stimuli. The main findings of the current study are (i) that anticipation of monetary punishments enhances long-term memory for punishment-predictive items, (ii) that dopamine genotype modulates recognition memory for motivational stimuli, and (iii) that dopamine genotype modulates striatal activity to reward and punishment anticipation and striatal and hippocampal activity related to subsequent memory for motivational items. These data are consistent with our hypothesis that dopaminergic action in the hippocampus is associated with higher memory for stimuli eliciting appetitive and aversive motivational processes.

Memory for punishment-predictive stimuli was enhanced in comparison to neutral stimuli. Such an effect of incentive motivation has previously been shown for reward-associated stimuli (Adcock et al., 2006; Callan & Schweighofer, 2008; Krebs et al., 2009; Wittmann et al., 2005; Wittmann, Dolan, & Düzel, 2011). In the context of reward, memory enhancement has been suggested to be mediated by dopamine release in the hippocampus. This is supported by data showing that hippocampal dopamine is necessary for the late phase of long-term potentiation, which it prolongs and enhances (Frey, Matthies, & Reymann, 1991; for a review see Lisman et al., 2011; O’Carroll & Morris, 2004; Sajikumar & Frey, 2004). In humans, integrity and activity of the dopamine system is correlated with individual memory performance (Backman et al., 2000; Cervenka et al., 2008; Takahashi et al., 2007, 2008). Consistent with these data, punishment-related and reward-related memory enhancement in the current study were stronger in DAT 10-repeat homozygotes compared to DAT heterozygous participants. Successful encoding of punishment-predictive items was associated with higher midbrain, striatal and hippocampal activity, supporting the idea that punishment anticipation elicited dopaminergic activity and thereby increased hippocampal encoding of cue stimuli. Successful encoding of reward-predictive items was associated with higher activity in striatum and hippocampus, but not in SN/VTA. Why there was an absence of a SN/VTA subsequent memory effect in the reward condition is unclear, since previous studies reported DM effects in SN/VTA both in motivational and non-motivational paradigms (Schott et al., 2004; Wittmann et al., 2005). However, another study that presented appetitive and aversive pictures also did not find this reward effect (Wittmann et al., 2008), suggesting that it could be influenced by the inclusion of an aversive category. In line with previous findings (Wittmann et al., 2005, 2008), activations in this study were located in ventral striatum and anterior hippocampus.

Previous studies investigating the effect of aversive motivation on memory reported inconsistent effects. In a human version of the Morris water maze (Murty et al., 2011), aversive electrical stimulation at incorrect platforms impaired learning. Memory was improved, however, when cues indicated aversive electrical stimulation if the current stimulus on display was not remembered at test 24 h later (Murty et al., 2012). This effect was mediated by amygdala-hippocampal interactions during the study phase of the task. The different network in comparison to the current study could result from the use of an intentional memory task, where punishment avoidance was dependent on successful memory formation. It is also possible that primary punishment such as shocks depends more strongly on an amygdala-based emotional system than secondary motivation elicited by monetary losses. This interpretation is supported by the finding that in an intentional memory task, threat of monetary loss enhanced source memory performance in an immediate memory test via a retrieval network of striatum and hippocampus (Shigemune et al., 2013), although fMRI results of the encoding phase were not reported. A behavioural study, in contrast, found no effect of punishment cues on immediate incidental memory for items presented as subsequent targets (Mather & Schoeke, 2011). Instead, the motivational effect on memory depended on the motivational outcome of each trial. This difference could be due to the 2–4 s delay between cue and encoded item, which would prevent dopaminergic modulation of hippocampal processing because of the phasic nature of dopaminergic activity to motivational cues. Another crucial difference between the two studies is the probability of punishment indicated by the cue, which was approx. 65% in the current study in contrast to a rate of approx. 33% in Mather and Schoeke (2011). In the context of the current study, therefore, results are consistent with the hypothesis that the high punishment probability indicated by the cues elicited phasic dopaminergic activity associated with the items at encoding and increased long-term memory via a striatal-hippocampal network.

The DAT1 VNTR polymorphism has been shown to affect expression of the dopamine transporter. The 9-repeat allele was found to be associated with higher levels of DAT in vivo (van de Giessen et al., 2009; van Dyck et al., 2005), while a post-mortem study reported the reverse association (Mill, Asherson, Browes, D’Souza, & Craig, 2002). Results from in vitro and in vivo studies have so far remained inconsistent (D’Souza & Craig, 2008; VanNess, Owens, & Kilts, 2005), and the impact of the DAT1 polymorphism on dopamine transmission cannot reliably be inferred. If we assume a higher DAT expression in 9-repeat carriers (van de Giessen et al., 2009; van Dyck et al., 2005), dopamine levels would be expected to be higher in 10-repeat homozygotes, which is supported by the higher activation in the reward system in homozygous participants in the current study. In contrast, several previous functional imaging studies found that 9-repeat carriers showed higher striatal activity in reward tasks (Aarts et al., 2010; Dreher, Kohn, Kolachana, Weinberger, & Berman, 2009; Forbes et al., 2009), while one study reported that genotype effects depended on an interaction with the personality trait reward sensitivity (Hahn et al., 2011). Differences between these studies could be due to variations in task protocols. Additional insight could be gained from studies that investigate the cumulative impact of multiple dopaminergic polymorphisms (e.g. Nikolova, Ferrell, Manuck, & Hariri, 2011; Stice, Yokum, Burger, Epstein, & Smolen, 2012) and interactions between several polymorphisms (e.g. Balci, Wiener, Cavdaroglu, & Branch Coslett, 2013; Dreher et al., 2009), although the inconsistent literature on each individual genotype currently still presents some difficulties for this approach. In the context of the current study, however, DAT genotype had consistent effects on anticipation of motivational outcomes and on memory for motivational outcomes. The current findings therefore support the possibility that dopamine contributes to striatal processing during anticipation of punishment and to hippocampal processing related to memory formation for motivational items.

A number of recent studies found activity in the dopaminergic system related to punishment. In anesthetized rats, dopamine neurons in the ventral VTA are excited by the onset of footshocks, whereas neurons in the dorsal VTA are inhibited by noxious stimulation (Brischoux et al., 2009). In monkeys, midbrain dopamine neurons respond to aversive air puffs by an increase in firing rate (Joshua, Adler, Mitelman, Vaadia, & Bergman, 2008; Matsumoto & Hikosaka, 2009), and there are indications that the neuronal populations coding for appetitive and aversive events are spatially separated (for a review see Bromberg-Martin et al., 2010). Analysis of distinct subpopulations within the SN/VTA was not possible in the current study given the spatial resolution of our fMRI protocol. The block design in combination with event-related within-block contrasts of motivational and neutral items, however, enabled us to eliminate factors that potentially confound findings of aversive dopaminergic responses: The contrast with neutral items from the same block eliminates general effects of a rewarding or punishing context on responses to all stimuli presented in that context. A generalized carry-over of neuronal responding from rewarded to punished items is additionally prevented by the temporal separation into rewarding and punishing blocks and by excluding the first three motivational and first three neutral stimuli in each block from the analysis, corresponding to about one minute at the beginning of each block. Although we cannot completely rule out the possibility of carryover effects, our data are consistent with other reports that have eliminated this confound behaviourally (Joshua et al., 2008; Matsumoto & Hikosaka, 2009) and with fMRI studies reporting an involvement of the striatum in active avoidance (Guitart-Masip et al., 2012, 2011; Levita, Hoskin, & Champi, 2012). We observed activation in the dopaminergic system to punishment cues but not outcomes. In line with previous results, this supports the conclusion that punishment-related activity is not caused by relief after termination of an aversive stimulus (Brischoux et al., 2009; Jensen et al., 2003; Matsumoto & Hikosaka, 2009), and suggests that neural activity was specific to the anticipation of motivational trials. This is also supported by the absence of striatal activation to reward compared to neutral or punishment outcomes, which is in line with the coding of prediction errors by the striatum (Garrison, Erdeniz, & Done, 2013). Since both reward and punishment were largely predicted by the cues in our task, we did not expect significant outcome-related activity in the dopaminergic system.

The DAT results support the hypothesis that striatal activation in fMRI studies on punishment is modulated by dopaminergic processes. Recent models of incentive learning and experimental findings suggest that the involvement of the dopaminergic system in punishment signals is related to action requirements (Boureau & Dayan, 2011; Guitart-Masip et al., 2011, 2012). In the current study, participants were instructed to try to avoid punishments by fast button presses. Although the staircase procedure ensured that the punishment rate remained above 65%, thus making it impossible to effectively avoid punishments, the lower reaction times in punishment compared to neutral trials indicate that participants were highly motivated and applied considerable effort towards punishment avoidance. The involvement of dopamine in punishment avoidance is hypothesized to depend on a shift of the motivational baseline in aversive contexts (Boureau & Dayan, 2011). In the current study, reward and punishment were presented in separate, alternating blocks. It is therefore possible that an adjustment of the baseline anticipation contributed to the striatal punishment signal. However, even in a punishment context, neutral trials would be expected to elicit more positive anticipation signals than punishment trials. The higher striatal activation to punishment cues in comparison to neutral cues can therefore not be fully explained by a shift in the motivational baseline. Our data suggest that striatal dopamine signals are equally involved in the anticipation of appetitive and aversive events when action is required to either obtain a reward or avoid a punishment (Guitart-Masip et al., 2011, 2012).

In conclusion, the present study showed that anticipation of monetary punishments and anticipation of monetary rewards exhibit overlapping patterns of neural activity and of long-term memory modulation. These effects were influenced by dopamine transporter genotype, suggesting that dopamine is involved in aversive motivation in humans. Our results provide support to the idea that dopamine enhances human long-term memory via a midbrain-striatal-hippocampal network and extend this network to include processing of punishment incentives.

## Figures and Tables

**Table 1:** Peak coordinates and fMRI statistics.

**Contrast**	**Region**	**MNI coordinates in mm**			**Statistics**		**Cluster size**
		*x*	*y*	*z*	Equivalent *Z*-value	No. of resels in SVC	Active voxels in SVC
See Fig. 3A	Putamen	11	10	−2	4.42	30.1	428
		11	16	1	4.09		
		7	12	−1	4.03		
		13	14	−7	3.97		
	SN/VTA	6	−22	−17	3.75	3.8	25
See Fig. 3B	Putamen	−11	11	−10	3.98	30.6	41
See Fig. 4A	Putamen	−3	9	−2	4.75	30.1	572
See Fig. 4B	Putamen	−16	12	−4	3.58	30.3	66
	Hippocampus	36	−16	−13	3.73	9.0	69
See Fig. 4C	Putamen	−24	1	6	4.11	31.9	260
	Hippocampus	25	−1	−21	3.49	9.5	38
See Fig. 4D	Putamen	−5	−2	−9	3.54	27.9	109
	Hippocampus	−15	−11	−20	3.74	8.3	146
		41	−11	−21	4.00		96
	SN/VTA	−4	−20	−14	3.79	3.5	87

*Note*: There were no significant activations outside the areas of interest at *p*<0.05 FWE-corrected.
